# Oral health care of people with Angelman syndrome in Germany – a questionnaire-based study

**DOI:** 10.1186/s12903-025-06357-9

**Published:** 2025-06-25

**Authors:** Peter Schmidt, Caroline Tantzen, Oliver Fricke, Andreas Gerhard Schulte

**Affiliations:** 1 Department of Special Care Dentistry, Witten/Herdecke University, Alfred-Herrhausen-Strasse 50, 58448 Witten, Germany; 2 Faculty of Health, Department of Medicine, Witten/Herdecke University, Alfred-Herrhausen-Straße 50, 58455 Witten, Germany; 3 Department of Child and Adolescent Psychiatry and Psychotherapy, Klinikum Stuttgart, Prießnitzweg 24, 70174 Stuttgart, Germany; 4 Department of Conservative Dentistry and Periodontology, Centre of Dentistry, Oral and Maxillofacial Medicine, Ulm University Hospital, University of Ulm, Albert-Einstein-Allee 11, 89081 Ulm, Germany

**Keywords:** Special care dentistry, Tooth brushing behavior, Assisted oral care, Caries prevention, Angelman syndrome, Dental health care

## Abstract

**Background:**

Caregivers of people with Angelman syndrome (AS) in Germany were surveyed to amend a lack of information on supportive and preventive oral care for persons in this group.

**Methods:**

Returned anonymized questionnaires that had been sent to the approx. 600 members of the German Angelman Syndrome Association were evaluated. The study was approved by the ethics committee of Witten/Herdecke University (# 121/2021).

**Results:**

In total, 220 questionnaires for people with AS aged between 1 and 54 years old (mean age 17.0 years) were evaluated. Overall, 38.1% (*n* = 84) of the people with AS were younger than three years at their first dental appointment; 60.0% (*n* = 132) tooth brushed twice daily; 15.9% (*n* = 35) brushed for 2–3 min; and 78.5% (*n* = 172) did not use dental hygiene products other than toothbrushes. Age-specific differences emerged: Although only 45.0% (*n* = 45) of people with AS ≥ 18 years (*n* = 100) began tooth brushing in the first year of life, this increased to 69.7% (*n* = 89) for people with AS < 18 years (*n* = 119). Also, while 76.5% (*n* = 91) of people with AS < 18 years were usually assisted with tooth brushing by the same person, this applied to only 50.0% (*n* = 50) of people with AS ≥ 18 years (*p* < 0.001; Chi-Square-Test).

**Conclusions:**

There are age-dependent differences in tooth brushing behavior among people with AS. Irrespective of age and sex, nearly all people with AS required life-long oral health support at home. Professional oral health support, e.g., regular check-ups, individual prophylactic measures, and dental cleaning, remains essential for this group. Hence, efforts must be increased to develop and offer more interprofessional dental prophylaxis concepts for people with AS of all ages, from infancy to senescence. These findings accord with those for other groups of people with syndromic disorders, such as Down syndrome.

**Clinical trial number:**

Not applicable.

**Supplementary Information:**

The online version contains supplementary material available at 10.1186/s12903-025-06357-9.

## Introduction

In 1965, the English pediatrician Dr. Harry Angelman (1915–1996) first published a scientific article in which he described the similar behavioral and clinical characteristics he had observed in three children [1]: “Their flat heads, jerky movements, protruding tongues and bouts of laughter give them a superficial resemblance to puppets, an unscientific name but one which may provide for easy identification” [1]. These characteristics led him to assume a syndromic disease, which Angelman described as “puppet syndrome” at the time. Later on, the syndrome was named after him and is, to this day, still known as Angelman syndrome (AS). Angelman syndrome (Q93.5, ICD-10 coding; LD90.0, ICD-11 coding) is a rare neurogenetic disorder that occurs due to an alteration on the maternal chromosome no. 15. Although the exact prevalence of the syndrome is unknown, it is said to occur in approximately 1:15,000 newborns of both sexes [2]. Various syndrome-related clinical features of Angelman syndrome (AS) can always be observed and can, therefore, be regarded as diagnostic symptoms. An updated consensus from 2005 thus identifies three diagnostic characteristics: (A) severe developmental delay, (B) disorders of movement and balance, especially ataxic gait and/or jerky movements of the extremities and (C) behavioral specificities such as frequent smiling/laughing; marked cheerfulness; mild irritability accompanied by (raised) hand clapping or waving, or hypermotoric behaviors [3]. Babies with Angelman syndrome, for example, twitch their hands and feet from early infancy on. In addition, various syndrome-related manifestations can also be seen in the orofacial and dental region, such as an elongated, narrow facial shape, mandibular prognathism with potential progeny and a wide mouth, in the sense of macrostomia. Macrostomia is often accompanied by teeth that are both gapped and widely spaced, which, in turn, may be reduced in shape or appear as such (supplementary file 2). Moreover, a number of oral motor behaviors with excessive oral activity are considered typical of AS. Some of these oral behaviors, such as “putting objects in the mouth” or “chewing on them” often last until puberty.

The characteristics of this syndrome exemplify why dental and oral health plays an important role in the daily lives of people with AS and why this group of people is dependent on the support of their social environment for care of this kind. Unfortunately, there is currently a worldwide lack of information on home-supported and professionally assisted oral hygiene for people with Angelman syndrome (AS). Hence, the main objective of the present study was to shed light on the tooth brushing behaviors, supportive oral care interventions, and use of caries-preventive fluoride products in children, adolescents, and adults with Angelman syndrome (AS) in Germany. A secondary objective was to investigate possible age- or sex-related differences (i.e., children and adolescents vs. adults; male vs. female) in this connection. A final objective was to compare the results for this group of people with those from studies on other groups of people with syndromic conditions (e.g., Down syndrome).

## Materials and methods

The present study was conducted in collaboration with the *Angelman e.V*., a German Angelman Syndrome association, which includes members living in Austria and Switzerland. The members of this nationwide, non-profit association (“Angelman e.V.”) are primarily family members of a person with AS. Both the members and the board of the association agreed to actively participate in this study.

This study follows the methodology and procedures of a previous published study, conducted with the German self-representation group for people with Down syndrome, published by Schmidt et al. 2022 [4]. The original source may be consulted for detail not provided in following summary.

### Questionnaire

A specifically designed paper-and-pencil questionnaire, which consisted of 133 questions (128 closed and 5 open) on the provision of oral health care, was used to collect the data. This questionnaire was devised in collaboration with the *Angelman e.V.* Association. Among these questions were 18 questions related to sociodemographic aspects and other ones that investigated the following aspects in regard to people with AS.

- tooth brushing behavior (7 questions),
- assisted oral health care at home and supportive oral hygiene interventions by dental practitioners (5 questions),
- use of fluoride, at home (5 questions) and.
- use of dental services (dentist) (1 question).

The present study evaluated the responses to these 36 questions from the returned questionnaires. The questions are attached as a supplementary file (supplementary file 1).

### Survey period and ethical aspects

As in our other studies [4, 5], anonymous survey questionnaires were sent to the 600 members/families of the *Angelman e.V.* Association, via the administration of the *Angelman e.V.* The questionnaires were dispatched in July 2021, followed by a written reminder in October 2021 that the deadline for the return of the completed questionnaires was the end of January 2022. Together with the questionnaire all members received an information letter about the procedure, the intention, etc. of the study. Due to the anonymous procedure, that was not appropriate to send an informed consent paper to be signed by a caregiver, because this would no longer have ensured anonymization. Thus sending a completed questionnaire to the study office constitutes an active decision to participate. The study was approved by the ethics committee of the Witten/Herdecke University (# 121/2021).

### Transfer of data, and statistics

The data were digitized and processed using Excel 2016 (Microsoft Corp., Redmond, Washington, DC, USA). Exploratory statistics were performed with SPSS Version 29 (IBM Corporation, New York, NY, USA). The data were manually entered and verified, independently, by two study team members (C.T. and P.K.). The arithmetic mean, median (age), minimum, maximum, frequency distributions (standard deviation), and significance were determined for the data. The Chi-square test (*p* ≤ 0.05) was used to explore differences between subgroups, which were formed by stratifying datasets by age (< 18 years old/≥18 years old) or sex (male/female).

In the following, people with Angelman syndrome are also referred to as the study population. People who completed a questionnaire for a family member with Angelman syndrome are referred to as parents or caregivers representing the study participants, e.g., siblings, other family members, or state-appointed professional guardians.

## Results

In total, 220 completed questionnaires were returned by parents or caregivers of people with AS living in Germany. This corresponds to a response rate of 36.7%. Largely, the questionnaires were completed (97.3%; *n* = 214) by parents of a person with AS. For minors, this percentage was 97.5% (*n* = 116) (Table 1). The study population (people with AS) included 107 males and 113 females, aged between 1 year and 54 years.

In all, 119 of the questionnaires (54.1%) were completed by parents or caregivers whose family members with Angelman syndrome were between 0 and 17 years old. In the age group of children or adolescents (0 to 17 years), there were 65 girls with AS and 54 boys with AS. Their mean age was 8.8 years (SD ± 4.75) and the age distribution was normally distributed. For one person with AS, no age information was given. The other 100 questionnaires provided information on adult people with AS (shown in Table 2).

**Fig. 1:**
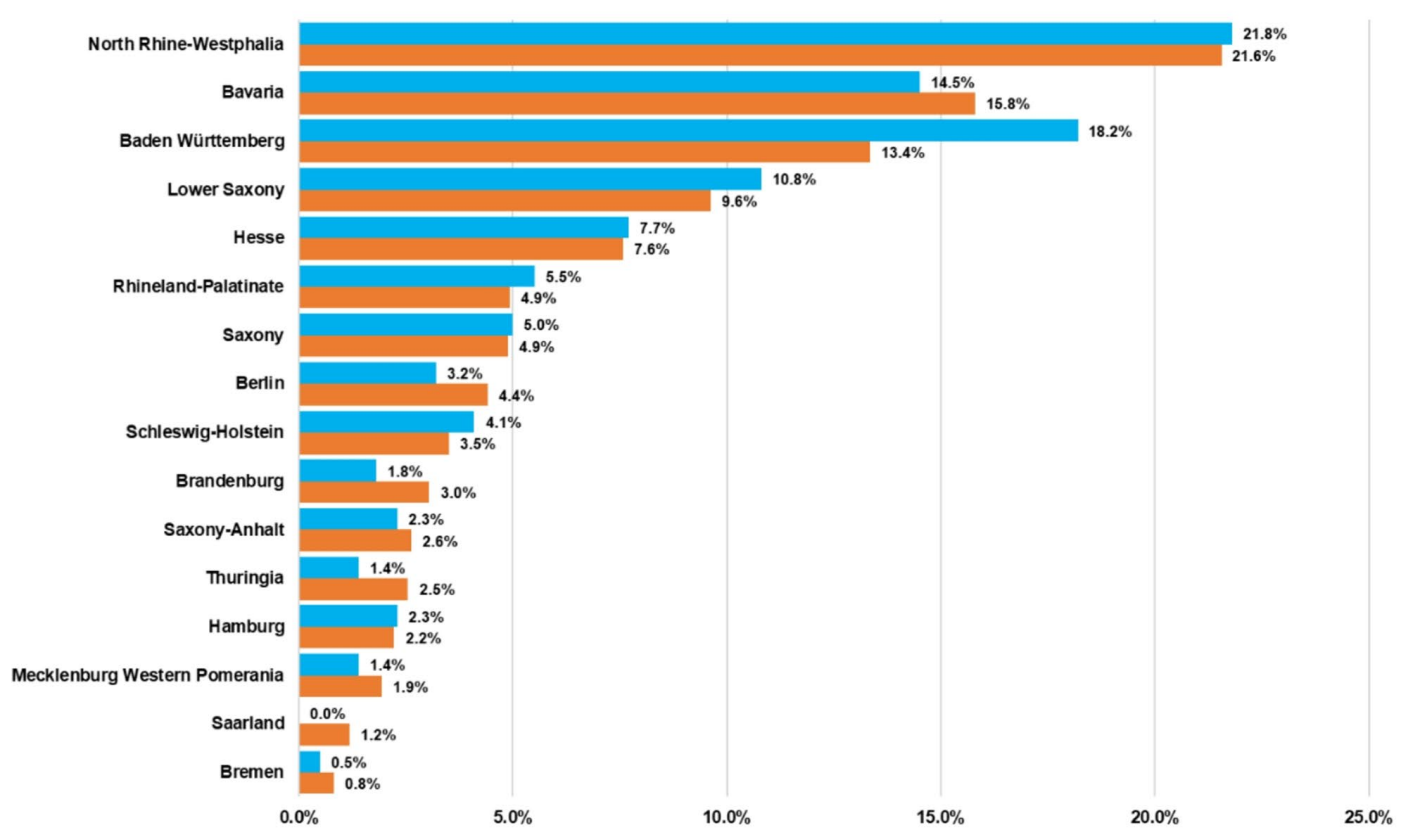
Comparison of the percentage distribution of inhabitants per German federal state in 2020 (orange), for a total population of 83.2 million people, with the percentage distribution of the study population per federal state (blue), for a total of 220 participants

Data could be collected from nearly all German federal states. During the survey period in 2021, the total population in Germany numbered 83.2 million people [6]. Figure 1 shows the proportional distribution of the study participants in relation to the number of inhabitants in the respective German federal states. By and large, the percentage distribution of participants corresponded with the percentage distribution of the German population (83.16 million) [6] across the 16 German federal states.

**Fig. 2:**
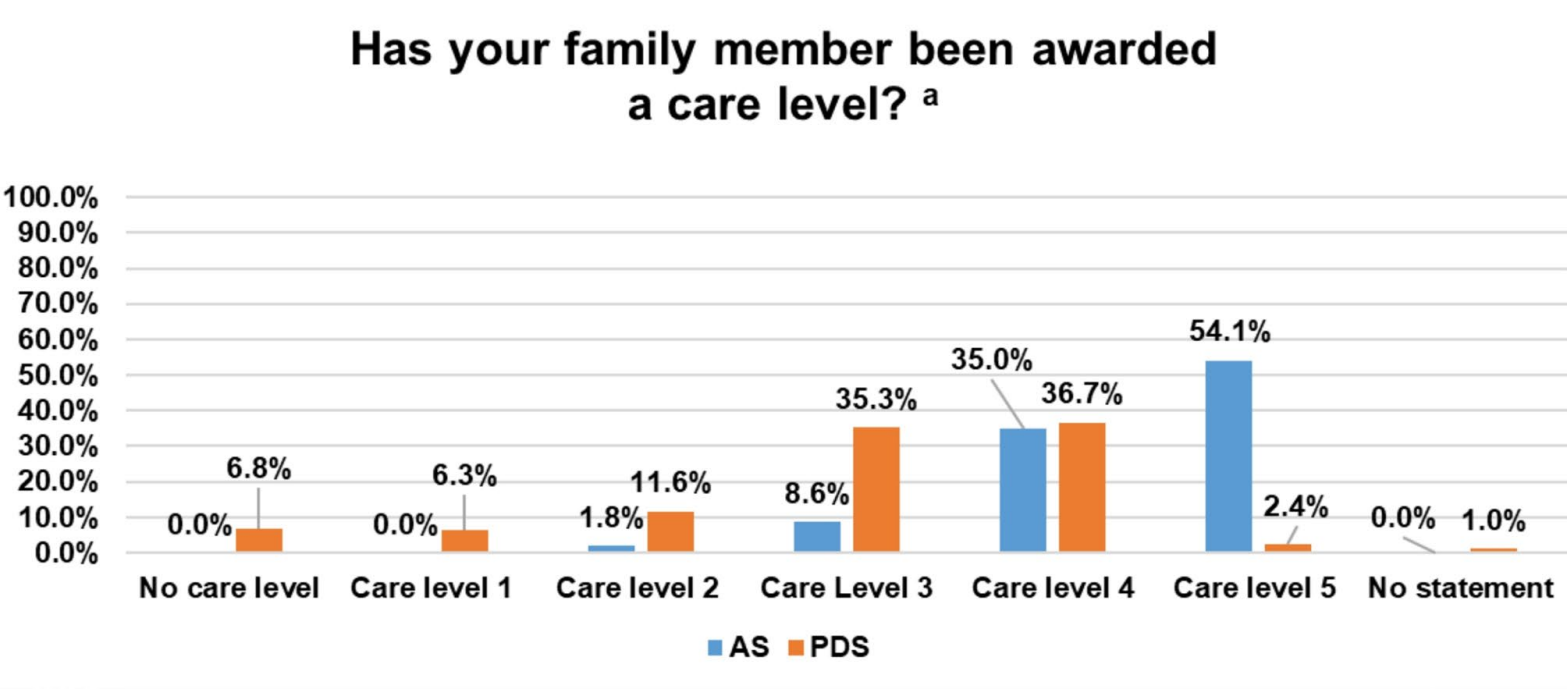
Information about the care level of people with Angelman syndrome (AS) vs. people with Down syndrome (PDS)

The majority of people with AS had lived in Germany since birth (*n* = 216; 98.2%). Irrespective of age and sex, most people with AS (*n* = 171; 77.8%) lived with their parents or other family members. In addition, more than half of the people with AS had been awarded the highest care level (level 5). In Germany, there are five care levels (1 to 5), which describe the different degrees of need for care and enable long-term care insurance benefits. The care levels are based on the individual’s level of independence and are determined on the basis of a care assessment. The higher the care level, the higher the entitlement to care benefits. A care level of 5 means that there is a severe level of disability with special care requirements. Table 2; Fig. 2 present further details on the study population.

**Table 1:** Characteristics of the respondents (family member/caregiver of the people with AS)

Characteristics of People Who Completed the Questionnaire (Study Participants)		
**Who completed the questionnaire?** (For all people with AS, n = 220)*1		
one parent	205	93.2%
both parents	10	4.5%
a family member (non-parent)	4	1.9%
a legal guardian	0	0.0%
a caregiver	0	-
other (free text response)	0	-
no statement	1	0.5%
Relationship to person with people with AS*		
mother	201	91.4%
father	24	10.9%
other relationship	4	1.9%
no statement	1	0.5%
**Age (in years)**		
mean ± SD median range (*n* = 216; *n* = 4 no statement)	48.4 ± 10.93 50 22–87	
**Who completed the questionnaire?** **(For all < 18 years old people with AS n = 119)**		
one parent	113	95.0%
both parents	3	2.5%
a family member (non-parent)	3	2.5%
a legal guardian	0	0.0%
a caregiver	0	0.0%
other (free text response)	0	0.0%
no statement	0	0.0%
**Age (in years)**		
mean ± SD median range (*n* = 118; *n* = 1 no statement)	41.1 ± 7.58 40 22–59	
**Who completed the questionnaire?** **(For all ≥ 18 years old people with As n = 100)**		
one parent	90	90.0%
both parents	7	7.0%
a family member (non-parent)	2	2.0%
a legal guardian	0	0.0%
a caregiver	0	0.0%
other (free text response)	0	0.0%
no statement	1	1.0%
**Age (in years)**		
mean ± SD median range (*n* = 97; *n* = 3 no statement)	57.2 ± 7.17 57 38–87	

* multiple responses possible, because in these questions more than one answer option could be chosen as well as one; a = *p* < 0.05; Chi-Square-Test

**Table 2:** Characteristics of the study population (People with AS)

People with Angelman syndrome (AS)	Frequencies	*n* = 220
**Sex**		
Male	107	48.6%
Female	113	51.4%
**Age (in years)**		
mean all ± (SD) median all range (*n* = 219; *n* = 1 no statement)	17.0 ± 10.73 16.00 1–54	
mean all (male) ± (SD) median all (male) range (male, *n* = 106; *n* = 1 no statement)	18.4 ± 11.00 17.0 2–54	
mean all (female) ± (SD) median all (female) range (female, *n* = 113)	15.7 ± 10.35 14 0.75–44	
mean all < 18 years ± (SD) median all (< 18 years) range (*n* = 119)	8.8 ± 4.75 8 0.75–17	
mean male < 18 years ± (SD) median male (< 18 years) range (male, *n* = 54)	9.6 ± 4.87 9 2–17	
mean female < 18 years ± (SD) median female (< 18 years) range (female, *n* = 65)	8.1 ± 4.58 7 0.75–17	
mean all ≥ 18 years ± (SD) median all (≥ 18 years) range (*n* = 100)	26.8 ± 7.02 25 18. − 54	
mean male ≥ 18 years ± (SD) median male (≥ 18 years) range (male, *n* = 52)	27.6 ± 7.55 27 18–54	
mean female ≥ 18 years ± (SD) median female (≥ 18 years) range (female, *n* = 48)	25.9 ± 6.37 23.5 18–44	
**Legal guardian**		
with guardian	102	46.4%
without guardian	4	1.8%
has no legal guardian (the person with AS is under 18 years old and the parents have the custody)	113	51.7%
no statement	1	0.5%
Living situation*		
alone or in shared accommodation or with a partner	4	1.8%
at parents’ home/at a family member’s home	171	77.8%
in supervised living	46	20.9%
other	5	2.3%

* multiple responses possible, because in these questions more than one answer option could be chosen as well as one; a = *p* < 0.05; Chi-Square-Test

### Tooth brushing behavior and assisted oral health care at home

The evaluation of the questionnaires showed that 58.2% (*n* = 128) of the people with AS had begun to receive regular dental hygiene care at home before their first birthday. For another 6.8% of the people with AS, regular dental hygiene care had begun at the age of two years or older (Table 3). Only one parent reported that the people with AS in their family could clean their teeth on their own, without assistance (0.5%). The vast majority of the people with AS (90.0%; *n* = 198), consistently needed assistance in cleaning their teeth. Of these, nearly two thirds of the people with AS were consistently assisted by the same caregiver, while the other third were regularly assisted by several caregivers. Overall, children and adolescents (76.5%; *n* = *9*1) were more frequently assisted by the same caregiver than adults (50.0%; *n* = 50). This difference was statistically significant (*p < 0.001*). In regard to tooth brushing frequency, 60.0% (*n* = 132) of the people with AS performed this task twice daily, with nearly identical percentages found across subgroups (i.e., male/female and children/adults). The majority of the people with AS (75.2%; *n* = 166) brushed their teeth for less than 2 min; only slightly less than one-fifth of the people with AS (15.9%; *n* = 35) brushed their teeth for 2–3 min. No differences emerged between the subgroups in regard to the type of toothbrush used (Table 3).

While 78.5% (*n* = 172) of the people with AS did not use dental hygiene implements other than toothbrushes, 6.8% (n = 15) of the people with AS used dental floss to clean interdental spaces, and 3.7% (*n* = 8) used interdental brushes. These findings were independent of age and sex.

No significant difference in acceptance was found between the two age groups (*p* = 0.291) or sexes (*p* = 0.126) in respect to toothpaste types. Almost two-thirds (62.3%; *n* = 137) of the surveyed people with AS accepted nearly all toothpaste types available on the German market (Table 3). The choice of toothpaste rested primarily on fluoride content or flavor. Table 3 provides further details on tooth brushing behavior.

**Table 3:** Tooth brushing preferences and fluoride use for people with Angelman syndrome (AS), as reported by the respondent

	**Children and Adolescents**	Adults	**Female**	**Male**	All people with AS
	(*n* = 119)	(*n* = 100)	(*n* = 113)	(*n* = 107)	(*n* = 220)
At what age did tooth brushing start for your family member with Angelman					
syndrome?					
• in the first year of life	69.7% (83) ^a^	45.0% (45) ^a^	61.1% (69)	55.1% (59)	58.2% (128)
• at the age of 1 year	23.5% (28	33.0% (33)	26.5% (30)	29.9% (32)	28.2% (62)
• at the age of 2 years	3.4% (4)	9.0% (9)	3.5% (4)	8.4% (9)	5.9% (13)
• at the age of 3 years	0.8% (1)	1.0% (1)	1.8% (2)	0.0% (0)	0.9% (2)
• I/we do not remember (anymore)	1.7% (2)	10.0% (10)	6.2% (7)	4.7% (5)	5.5% (12)
• no statement	0.8% (1)	2.0% (2)	0.9% (1)	1,9% (2)	1.4% (3)
Does your family member with Angelman syndrome receive assistance in tooth					
brushing?					
• brushes teeth alone	0.0% (0)	1.0% (1)	0.0% (0) ^a^	0.9% (1) ^a^	0.5% (1)
• needs assistance with tooth brushing	90.8% (108)	89.0% (89)	85.8% (97)	94.4% (101)	90.0% (198)
• receives assistance with tooth brushing	10.0% (2)	3.0% (3)	4.4% (5)	0.0% (0)	2.3% (5)
• receives assistance with tooth brushing at least once a week	0.0% (0)	0.0% (0)	0.0% (0)	0.0% (0)	0.0% (0)
• receives assistance with tooth brushing at least once a day	7.6% (9)	7.0% (7)	9.7% (11)	4.7% (5)	7.3% (16)
Who assists with tooth brushing?					
• usually the same person	76.5% (91) ^a^	50.0% (50) ^a^	63.7% (72)	65.4% (70)	64.5% (142)
• almost always several persons	23.5% (28)	49.0% (49)	36.3% (41)	33.6% (36)	35.0% (77)
• does not apply	0.0% (0)	0.0% (0)	0.0% (0)	0.0% (0)	0.0% (0)
• no statement	0.0% (0)	1.0% (1)	0.0% (0)	0.9% (1)	0.5% (1)
How often does your family member with Angelman syndrome usually brush					
teeth? *					
• once a day	38.7% (46) ^a^	32.0% (32) ^a^	38.1% (43)	34.6% (37)	36.4% (80)
• twice a day	58.8% (70)	61.0% (61)	58.4% (66)	61.7% (66)	60.0% (132)
• three times a day	2.5% (3)	4.0% (4)	2.7.% (3)	3.7% (4)	3.2.% 7)
• after every meal	0.0% (0)	1.0% (1)	0.9% (1)	0.0% (0)	0.5% (1)
• brushed teeth regularly several times a week	1.7% (2)	1.0% (1)	0.9% (1)	1.9% (2)	1.4% (3)
• teeth cannot be brushed regularly	1.7% (2)	2.0% (2)	1.7% (2)	1.9% (2)	1.8% (4)
• no statement	0.0% (0)	2.0% (2)	0.9% (1)	0.9% (1)	0.9% (2)
Please estimate how long your family member usually accepts tooth brushing?*					
• less than 1 min	41.2% (49) ^a^	24.0% (24) ^a^	30.1% (34)	37.4% (40)	33.6% (74)
• 1–2 min	40.3% (48)	44.0% (44)	48.7% (55)	34.6% (37)	41.8% (92)
• 2–3 min	14.3% (17)	18.0% (18)	11.5% (13)	20.6% (22)	15.9% (35)
• this is different every time	5.0% (6)	15.0% (15)	8.8% (10)	10.3% (11)	9.5% (21)
• no statement	0.0% (0)	2.0% (2)	1.8% (2)	0.0% (0)	0.9% (2)
What type of toothbrush does your family member with Angelman syndrome					
use for tooth brushing?					
• manual toothbrush	24.4% (29)	18.0% (18)	23.0% (26)	19.6% (21)	21.4% (47)
• electric toothbrush	38.7% (46)	40.0% (40)	38.1% (43)	40.2% (43)	39.1% (86)
• sonic electric toothbrush	3.4% (4)	7.0% (7)	7.1% (8)	2.8% (3)	5.0% (11)
• both manual and electric toothbrush	33.6% (40)	35.0% (35)	31.9% (36)	37.4% (40)	34.5 (76)
• other	0.0% (0)	0.0% (0)	0.0% (0)	0.0% (0)	0.0% (0)
	**Children and Adolescents**	**Adults**	**Female**	**Male**	**All people with AS**
	**(*n*** **= 119)**	**(*n*** **= 100)**	**(*n*** **= 113)**	**(*n*** **= 107)**	**(*n*** **= 220)**
Are additional dental hygiene implements used apart from the tooth-brush? *					
• Interdental brushes	0.0% (0) ^a^	8.0% (8) ^a^	2.7% (3)	4.7% (5)	3.7% (8)
• dental floss	5.1% (6)	9.0% (9)	6.2% (7)	7.5% (8)	6.8% (15)
• dental woods	0.0% (0)	0.0% (0)	0.0% (0)	0.0% (0)	0.0% (0)
• Tongue cleaner	0.8% (1)	3.0% (3)	2.7% (3)	0.9% (1)	1.8% (4)
• Oils	6.8% (8)	1.0% (1)	4.4% (5)	3.8% (4)	4.1% (9)
• no additional implements	82.2% (97)	74.0% (74)	77.0% (87)	3.8% (4)	78.5% (172)
• other additional implements	2.5% (3)	8.0% (8)	6.2% (7)	80.2% (85)	5.0% (11)
• no statement	3.4% (4)	2.0% (2)	3.5% (4)	0.9% (1)	2.3% (5)
Are any fluoride products used for caries prevention? *					
• mouth rinse with fluoride	0.0% (0) ^a^	6.0% (6) ^a^	3.5% (4)	1.9% (2)	2.7% (6)
• gels with fluoride	17.6% (21)	14.0% (14)	15.0% (17)	16.8% (18)	15.9% (35)
• table salt with fluoride	71.4% (85) ^a^	57.0% (57) ^a^	65.5% (74)	64.5% (69)	65.0% (143)
• no use of these fluorides	22.7% (27)	17.0% (17)	23.0% (26)	16.8% (18)	20.0% (44)
Did your family member receive fluoride tablets as a child?					
• yes	53.8% (64) ^a^	67.0% (67) ^a^	56.6% (64)	62.6% (67)	59.5% (131)
• no	41.2% (49)	20.0% (20)	34.5% (39)	29.0% (31)	31.8% (70)
• I/we do not remember (anymore)	4.2% (5)	11.0% (11)	8.0% (9)	6.5% (7)	7.3% (16)
• no statement	0.8% (1)	2.0% (2)	0.9% (1)	1.9% (2)	1.4% (3)

* multiple responses possible, because in these questions more than one answer option could be chosen as well as one; ^a^ = *p* < 0.05; Chi-Square-Test

### Use of household products with fluoride

Various household products that contain fluoride, which can help prevent dental caries, are available for domestic use in Germany. The survey questionnaire included questions in respect to three fluoride-containing product groups (Table 3). Although nearly two-thirds (65.0%; *n* = 143) of the responding parents/caregivers of all surveyed people with AS reported using fluoridated table salt at home for the preparation of meals, the use of fluoride mouth rinses (2.7%; *n* = 6) or fluoride gels (15.9%; *n* = 35) was not widespread. More detailed information, for instance, in respect to the two age groups, can be found in Table 3.

***The use of dental health services and professional supportive oral hygiene interventions***: Slightly more than half of the people with AS (57.8%; *n* = 127) had reportedly been older than two years at their first dental appointment. Another 4.1% (*n* = 9) of the persons who completed the questionnaire declared that their person with AS had never visited a dentist prior to the date on which the questionnaire was completed. Only 5.0% of the people with AS under study went to a dentist before the age of one year. In regard to the most recent visit to a dentist, the majority of the study population (all people with AS: 81.4%, *n* = 179; people with AS < 18 years: 84.0%, *n* = 100; people with AS ≥ 18 years: 78.0%, *n* = 78), cited check-ups as the underlying reason. Prophylaxis sessions were given as the reason by another 24.5% of the people with AS. Overall, the responses differed between the age groups (people with AS < 18 years: 13.4%, *n* = 16; people with AS ≥ 18 years: 38.0%, *n* = 38).

The survey also revealed that, irrespective of their age or sex, the majority of the people with AS (66.8%; *n* = 147) had not been instructed on how to brush their teeth in a dental practice, with only 6.4% (*n* = 14) of the parents/caregivers affirming that tooth brushing had been actively practiced with the person with AS in a dental practice. Table 4 presents further information on supportive oral hygiene interventions by dental providers.

**Table 4:** Information about professional dental care for people with Angelman syndrome (AS), as reported by the respondent

	Children and Adolescents	Adults	Female	Male	All people with AS
	(*n* = 119)	(*n* = 100)	(*n* = 113)	(*n* = 107)	(*n* = 220)
When was your family member’s first visit to the dentist?					
• in the first year of life	7.6% (9) ^a^	2.0% (2) ^a^	7.1% (8)	2.8% (3)	5.0% (11)
• at the age of 1 year	24.4% (29)	4.0% (4)	17.7% (20)	12.1% (13)	15.0% (33)
• at the age of 2 years	21.8% (26)	13.0% (13)	20.4% (23)	15.9% (17)	18.2% (40)
• at the age of 3–5 years	30.3% (36)	33.0% (33)	27.4% (31)	35.5% (38)	31.4% (69)
• at the age of 6–10 years	4.2% (5)	22.0% (22)	8.8% (10)	15.9% (17)	12.3% (27)
• I/we do not remember (anymore)	2.5% (3)	20.0% (20)	10.6% (12)	10.3% (11)	10.5% (23)
• not yet at all	6.7% (8)	1.0% (1)	3.5% (4)	4.7% (5)	4.1% (9)
• no statement	2.5% (3)	5.0% (5)	4.4% (5)	2.8% (3)	3.6% (8)
Were tooth brushing techniques explained and demonstrated to the family member at the dental practice?					
• yes	24.4% (29)	23.0% (23)	27.4% (31)	19.6% (21)	23.6% (52)
• no	65.5% (78)	69.0% (69)	64.6% (73)	69.2% (74)	66.8% (147)
• other	3.4% (4)	4.0% (4)	2.7% (3)	5.6% (6)	4.1% (9)
• no statement	6.7% (8)	4.0% (4)	5.3% (6)	5.6% (6)	5.5% (12)
Was tooth brushing practised with your family member at the dental practice you visited?					
• yes	8.4% (10)	4.0% (4)	7.1%(8)	5.6% (6)	6.4%(14)
• no	84.0% (100)	89.0% (89)	85.8% (97)	86.9% (93)	86.4% (190)
• other	2.5% (3)	3.0% (3)	1.8% (2)	3.7% (4)	2.7% (6)
• no statement	5.0% (6)	4.0% (4)	5.3% (6)	3.7% (4)	4.5% (10)
Were tooth brushing techniques also explained and demonstrated to the care- giver who assists the PAS with dental care at the dental practice you visited?					
• yes	27.7% (33)	26.0% (26)	30.1% (34)	23.4% (25)	26.8% (59)
• no	57.1% (68)	62.0% (62)	55.8% (63)	63.6% (68)	59.5% (131
• does not apply, as no supportive dental care is provided	4.2% (5)	6.0% (6)	5.3% (6)	4.7% (5)	5.0% (11)
• other	0.0% (0)	0.0% (0)	0.0% (0)	0.0% (0)	0.0% (0)
• no statement	10.9% (13)	6.0% (6)	8.8% (10)	8.4% (9)	8.6% (19)

* multiple responses possible, because in these questions more than one answer option could be chosen as well as one; a = *p* < 0.05; Chi-Square-Test

**Table 5:** Comparison of the tooth brushing preferences of people with Angelman syndrome (AS) with those of people with down syndrome (PDS) [4]

	**PDS [4]**	people with AS
	**(*n*** **= 207)**	**(*n*** **= 220)**
Does your family member receive assistance in tooth brushing? *		
• brushes teeth alone	60.4% (125) ^a^	0.5% (1) ^a^
• needs assistance with tooth brushing	38.6% (80)	90.0% (198)
• receives assistance with tooth brushing	18.4% (38)	2.3% (5)
• receives assistance with tooth brushing at least once a week	10.1% (21)	0.0% (0)
• receives assistance with tooth brushing at least once a day	18.8% (39)	7.3% (16)
Who assists with tooth brushing?		
• usually the same person	44.0% (91) ^a^	64.5% (142) ^a^
• almost always several persons	8.7% (18)	35.0% (77)
• does not apply	41.5% (86)	0.0% (0)
• no statement	0.0% (0)	0.5% (1)
How often does your family member usually brush teeth?		
• once a day	14.5% (30) ^a^	34.9% (80) ^a^
• twice a day	78.3% (162)	57.6% (132)
• three times a day	4.8% (10)	3.1% (7)
• after every meal	1.0% (2)	0.4% (1)
• brushed teeth regularly several times a week	0.0% (0)	1.3% (3)
• teeth cannot be brushed regularly	1.0% (2)	0.0% (0)
• no statement	0.5% (1)	0.9% (2)
Please estimate how long your family member usually accepts		
tooth brushing? *		
• less than 1 min	7.7% (16) ^a^	33.6% (74) ^a^
• 1–2 min	49.3% (102)	41.1% (92)
• 2–3 min	30.9% (64)	15.6% (35)
• this is different every time	10.6% (22)	9.4% (21)
• no statement	1.4% (3)	0.9% (2)
Does your family member accept almost all types of toothpaste?		
• he/she accepts almost all types of toothpaste	68.6% (142)	62.3% (137)
• he/she only accepts a few types of toothpaste	15.0% (31)	21.4% (47)
• I don't know / we don't know (anymore)	13.5% (28)	15.9% (35)
• no statement	2.9% (6)	0.5% (1)

* multiple responses possible, because in these questions more than one answer option could be chosen as well as one; a = *p* < 0.05; Chi-Square-Test

## Discussion

The present study, which collected national data for Germany through a postal questionnaire, is the first to allow insights into the tooth-brushing behavior and preferences of children, adolescents, and adults with Angelman syndrome, as well as into their use of fluoride, as a caries preventive measure, at home. The study also provided information in regard to the level of assisted oral health care provided to people with AS by parents/caregivers at home, the use of dental health services, and the accessibility of professional supportive oral hygiene interventions (e.g., advice, demonstration, and training of oral hygiene procedures) for persons with Angelman syndrome.

The present study is one of several studies on oral health care that have been conducted in Germany in recent years in collaboration with the self-representation groups of people with various disabilities/syndromes/rare diseases [4, 5, 7–9]. While studies on the oral health care of persons with Down syndrome can be found in the literature for various other global regions (e.g. Arabia, Asia, Europe, North America, South America), a study providing relevant data on persons with Down syndrome in Germany has only recently become available [4]. In contrast, only a few dental studies and reports are so far available on the oral health and dental treatment of people with Angelman syndrome. Explicitly, these are e.g., two case reports from Brazil, one case report from Italy, and one case report from Korea on dental therapies, including general anaesthesia [10–13]. In addition, the authors identified a study by Zilberman and Zilberman which focused on the effect of Angelman syndrome on enamel and dentin mineralization. In this study, Zilberman and Zilberman. describe effects of the syndrome on the morphology and mineralization of enamel, e.g., hypoplastic enamel and abnormal protein content in comparison to match-paired normal teeth [14]. To the best of our knowledge, however, no oral and dental health studies that are comparable with our present study have yet been published worldwide in relation to people wih AS (status: autumn 2024).

Based on our experience, the return rate of 36.7% for the questionnaires in our study was high for German standards. The response rate in the present study was, in fact, even higher than that in our published study on the oral health of people with Down syndrome in Germany [4]. Nonetheless, the response rate must still be seen as a limitation of the study, as it is conceivable that the respondents represent a different socio-demographic group than those who did not respond. The results might, thus, have been different if, e.g., 90% of the members had responded. Our research group has already previously experienced this limitation in the context of a study with people with Down syndrome. This limitation, in terms of low response rates, once again illustrates one of the scientific challenges encountered in conducting research in such population groups [4, 5]. Another limitation of our study is the absence of a control group, which could not be included due to organizational, data security, and financial reasons. Nevertheless, because one of the aims of our study was to survey families with a family member with AS of all ages and genders, and not only pre-specified sample groups, we believe that the data from our survey can still be considered representative: The participants with AS can be seen to encompass all age groups and both sexes in the appropriate distribution (Table 2). In addition, the percent distribution of the participants in our study largely reflects the percent distribution of inhabitants across the German federal states (Fig. 1).

We believe that a key factor in raising awareness for our study was our early collaboration with the “Angelman e.V.” in preparing the survey. Study participants were thus given the opportunity to suggest themes that they, themselves, considered relevant in respect to the provision of oral or dental care for people with AS. The numerous messages that were forwarded to the authors of this study from members of the “Angelman e.V.” in this context were deemed an indication of just how relevant the topic of dental care appears to be for families with a family member with Angelman syndrome. The circumstance that a survey on this topic was being conducted in Germany for the first time was also viewed very positively by many participants. The positive reception of the present study may also explain why all of the returned questionnaires were almost fully answered by the participants. The level of positive feedback in regard to the study topic, not only within the group of people with AS, but also from other syndromic population groups in similar studies–such as already published in relation to people with Down syndrome [4, 15]--is encouraging.

An important insight provided by the present survey is that only a segment of the parents of a person with AS (58.2%; *n* = 128) had begun to brush their children’s teeth regularly before the child’s first birthday. This finding is comparable to that found in our survey of people with Down syndrome, even if this percentage was slightly higher (66.6%; *n* = 138) in that group [4]. Interestingly, although this finding depended on the current age of the person with AS, it was independent of sex. To minimize stress in relation to oral and dental care, the authors strongly encourage the early introduction of regular oral hygiene routines into a child’s life, in the sense of babies and toddlers already becoming accustomed to the sensation of implements such as toothbrushes in their mouths. Hence, tooth brushing should be adopted as a routine procedure as soon as a baby’s first primary teeth erupt, even if dental health care and dental hygiene, understandably, do not rank foremost among the primary concerns of young parents during the first months or years of their child’s life. From their clinical experience, the authors of this study understand that, in light of the various other general health issues that may exist, this is particularly true for the parents of a child with a congenital disability. Topics such as dental health and dental appointments may then not seem of foremost importance. Encouragingly, however, the present data on people with AS seem to indicate that the importance of tooth brushing from an early age on is increasingly being acknowledged. In our view, the finding that the percentage of people with AS who started brushing their teeth in the first year of life was significantly higher in the group encompassing children and adolescents than in the adult group (Table 3) is a positive development, which also suggests a growing understanding of how to maintain oral health and prevent disease. This perception is also backed by the responses to our survey in regard to age at first dental appointments for people with AS: In nearly two of three cases (61.9%; *n* = 136) the people with AS were two years old or older at their first visit to the dentist (Table 4). This finding, again, was dependent on the current age of the people with AS: This time frame for a first dental visit was significantly more frequent in families with a family member with AS aged up to three, than in other families with a person with AS in childhood or adolescence (people with AS < 18 years: 53.8%, *n* = 64) or in adulthood (people with AS ≥ 18, 19.0% *n* = 19). The extent to which the use of dental professional services in dental practices, from an early age on, may contribute to improving the oral and dental health of people with AS cannot yet be described. To our best knowledge, there is also currently no available data in this respect in national or international literature. A few studies conducted on German toddlers without underlying diseases, however, demonstrate that timely visits to dentists during babyhood already can help prevent early childhood caries [16–18]. In our view, more such services and programs should be offered for groups of people with specific disabilities or syndromes, e.g. in combination with special dental consultations, and these should be scientifically monitored. This view is underscored by the findings of several international studies which show that people with disabilities, autism, rare diseases or syndromes have less access to oral health care [19–24].

Another important parameter of dental self-care in such groups is the frequency with which teeth are brushed and the length of time that tooth brushing is accepted. The current survey showed that, irrespective of their age and sex, nearly two thirds of the people with AS (60.0%) brushed their teeth twice daily (Table 3). Interestingly, according to the literature, the percentage of persons with cerebral palsy who brushed twice daily was considerably lower (30%) [25]. On the other hand, our study on people with Down syndrome showed that a higher percentage of individuals in that group brushed twice daily (78.3%) in comparison to the group of people with AS [4].

At this point, it should be noted that in addition to the frequency of tooth brushing, the duration should also be considered. Both theses aspects are also mentioned in German guidelines for caries prophylaxis in permanent teeth: In addition to brushing twice daily, teeth should also be brushed for the duration of at least two minutes, irrespective of the type of toothbrush used [26, 27]. Only 15.9% of the people with AS in our study, however, were estimated to be capable of brushing their teeth for two to three minutes (Table 3). By comparison, in our study on people with Down syndrome, we observed that the proportion of individuals capable of brushing for this length of time was almost twice as high (30.9%) as in the group in people with AS [4]. These distinctly different frequencies could be due, among other things, to the different clinical manifestations of both syndromes (Down and Angelman). As described at the beginning, the following three characteristics are typical of the Angelman syndrome: (A) severe developmental delay, (B) disorders of movement and balance, especially ataxic gait and/or jerky movements of the extremities and (C) behavioral specificities and mild irritability accompanied by (raised) hand clapping or waving or hypermotor behavior [3]. In contrast, the group of people with Down syndrome can be characterized as having a wide range of self-efficacy in regard to dental and oral care, depending on the degree of intellectual disability associated with the syndrome and the level of motor skills. As such, there may be people with Down syndrome who only have a moderate need for supportive dental and oral care. On the other hand, a subgroup of people with Down syndrome, across all age groups, may require more extensive support throughout their lives. This enormously wide range, e.g. in regard to intellectual and motor skills, does not appear to exist within the range of variance associated with AS. Consequently, daily dental and oral care in people with AS can be described as involving a high level of support and posing a challenge for their social environment. In this light, the responses to the question “Does your family member with Angelman syndrome receive assistance in tooth brushing?” appear plausible: According to their parents or caregivers, 90% of the people with AS in our study, irrespective of their age and sex, needed assistance with cleaning their teeth (Table 3). To provide context, 92.3% of children with cerebral palsy receive this kind of support [25, 28]. Based on our own clinical observations, the reported percentage of people with AS of all ages who need support with tooth brushing appears both plausible and representative.

Furthermore, the data on who assists with tooth brushing also confirms what we observe in the clinic. In the study, slightly over three quarters of the parents (76.5%) of people with AS younger than 18 years reported that usually the same person assisted the person with AS in their family with tooth brushing. In contrast, for the age group of people with AS older than 18 years, only 50.0% of parents indicated that this assistance was still regularly given by the same person (Table 3). Fundamentally, the age of the person with AS appears to be significant in regard to who assists them in cleaning their teeth. In the case of people with disabilities in adulthood, this finding may mainly be due to the fact that they often live in assisted living facilities, where (changing) caregivers are also responsible for dental and oral care. An interesting trend in this respect has been reported in the current literature from Germany: As we have also repeatedly pointed out in previous studies, the residential setting of people with impairments and disabilities represents a person-related parameter in relation to oral health. This parameter should definitely be further examined in future studies [29, 30]. In the national German DMS V study, similar observations were made for a group of elderly senior citizens in need of care. In regard to acute caries (DT value), the study found that study participants in need of inpatient care had an average caries prevalence that was around three times higher (1.3 teeth versus 0.5 teeth) than that for study participants who were cared for in their own homes [31].

In respect to the use of dental services, most parents in the current survey named check-ups or prophylaxis sessions as the reason for their most recent visit to a dentist with their family member with Angelman syndrome. Moreover, the responses were, by and large, similar for both age groups. These findings are also in accord with those reported for persons with Down syndrome living in Germany or other European countries [4, 32–35].

The use of fluorides is also one of various preventive measures to reduce dental caries. Although water fluoridation has not found acceptance in Germany, fluoridated salt was introduced in 1991 [36]. Quite generally, the use of fluoridated table salt has risen in Germany over the years and is now widely used [36]. Hence, almost two-thirds (65.0%) of the participants in the present study affirmed that they used fluoridated table salt at home. This percentage was even slightly higher for the people with AS in the childhood and adolescent age group (Table 3). Similar percentages for the use of fluoridated salt were reported in our other studies based on data from questionnaires among different self-representation groups of people with disabilities in Germany [4, 5]. Our findings in this regard are, therefore, in keeping with those for the general population in Germany, as can be seen from the data published by the current German epidemiological oral health study (DMS V) [31].

As described at the beginning of the discussion, people with AS are, in many cases, actively assisted with their daily oral hygiene procedures by caregivers or parents. It is, therefore, important to stress that both people with AS of all ages and their caregivers ought to be professionally instructed in proper tooth brushing techniques by dental practitioners within the scope of supportive oral hygiene interventions. The finding that such instruction had only been received by a quarter of the participating parents/caregivers of people with AS is unsatisfactory (Table 4). Moreover, only slightly over 6% of the respondents reported that proper tooth brushing had been actively practiced with the person with AS in a dental practice. This percentage was essentially the same for both sexes and both age groups (Table 5).

## Conclusions

The present study is the first, worldwide, to investigate oral health care and oral health behaviors in people with Angelman syndrome in collaboration with a national self-representation group for people with Angelman syndrome.

In some aspects, the tooth brushing habits and oral self-care behavior of people with Angelman syndrome, of all ages, differ from those of people with other syndromes, e.g. Down syndrome, or of those of people without disability. Moreover, within the group of people with Angelman syndrome, numerous age-related differences in tooth brushing behavior were found, such as the age at which tooth brushing began and who assisted with tooth brushing. Overall, there were no sex-related differences in this respect. Nearly all people with Angelman syndrome, irrespective of their age and sex, need life-long oral health support at home. In addition, professional oral health support, e.g., in the form of regular check-ups, individual prophylactic measures, and dental cleaning, is also essential for people with Angelman syndrome in all phases of their lives. Hence, there is need to increase the efforts in developing and offering more inter-professional dental prophylaxis concepts tailored to people with Angelman syndrome of all ages, from infancy to senescence. Personalized check-up intervals and hands-on education and training in oral hygiene through dental staff should form a part of these concepts. Ideally, such concepts would be transferable to different groups of people with other syndromes; for example, by flexibly including specific syndrome-related services.

## Electronic supplementary material

Below is the link to the electronic supplementary material.

Supplementary Material 1

Supplementary Material 2

## Data Availability

Due to the strict European General Data Protection Regulation and the statement in the questionnaire to the study participants, no data will be passed on to third parties. The dataset generated from this study cannot be deposited in a public repository, because the study participant consent did not include data sharing permissions. A request for access to data for researchers who meet criteria for access to confidential data must be made to the first author: Peter Schmidt, email: peter.schmidt@uni-wh.de, or to a representative of our Department of Special Care Dentistry, Dental School, Faculty of Health, Witten/Herdecke University, Germany and the board of the “Angelman e.V. ”, Germany, too.

## References

[1] Angelman H, ‘Puppet’ Children A, Report on Three Cases. Dev Med Child Neurol. 1965;7(6):681–8. 10.1111/j.1469-8749.2008.03035.x. 10.1111/j.1469-8749.2008.03035.x 18754889

[2] Bonello D, Camilleri F, Calleja-Agius J. Angelman syndrome: identification and management *Neonatal network: NN* 2017, 36(33):142–51. 10.1891/0730-0832.36.3.142 10.1891/0730-0832.36.3.142 28494826

[3] Williams CA, Beaudet AL, Clayton-Smith J, Knoll JH, Kyllerman M, Laan LA, Magenis RE, Moncla A, Schinzel AA, Summers JA, et al. Angelman syndrome 2005: updated consensus for diagnostic criteria. Am J Med Genet. 2006;140A:413–8. 10.1002/ajmg.a.31074. 10.1002/ajmg.a.31074 16470747

[4] Schmidt P, Suchy LC, Schulte AG. Oral health care of people with down syndrome in Germany. Int J Environ Res Public Health. 2022;19:12435. 10.3390/ijerph191912435. 36231733 10.3390/ijerph191912435 PMC9564659

[5] Kraus H, Schulte AG, Fricke O, Schmidt P. Tooth brushing behavior and oral health care of people with early childhood autism in Germany. Clin Oral Invest. 2025;29:112. 10.1007/s00784-025-06194-8. 10.1007/s00784-025-06194-8 PMC11799013 39907817

[6] Destatis. Bevölkerung - Anzahl der Einwohner in den Bundesländern in Deutschland am 31. Dezember 2020 Available online: https://de.statista.com/statistik/daten/studie/71085/umfrage/verteilung-der-einwohnerzahl-nach-bundeslaendern/#professional (accessed on 06.11.2024).

[7] Hanisch M, Blanck-Lubarsch M, Bohner L, Suwelack D, Kleinheinz J, Köppe J. Oral conditions and oral Health-Related quality of life of people with Ehlers-Danlos syndromes (EDS): A Questionnaire-Based Cross-Sectional study. Med (Kaunas). 2020;56. 10.3390/medicina56090448. 10.3390/medicina56090448 PMC7559544 32899664

[8] Niekamp N, Kleinheinz J, Reissmann DR, Bohner L, Hanisch M. Subjective oral health-Related quality of life and objective oral health in people with ectodermal dysplasia. Int J Environ Res Public Health. 2020;18. 10.3390/ijerph18010143. 10.3390/ijerph18010143 PMC7796382 33379169

[9] Steur J, Bohner L, Jackowski J, Hanisch M, Oelerich O. Oral health and oral-health-related quality of life in people with X-linked hypophosphatemia. BMC Oral Health. 2024;24:259. 10.1186/s12903-024-04028-9. 38383400 10.1186/s12903-024-04028-9 PMC10880295

[10] Murakami C, Nahás Pires Corrêa MS, Nahás Pires Corrêa F. Nahás Pires corrêa, J.P. Dental treatment of children with Angelman syndrome: a case report. Spec Care Dentist. 2008;28:8–11. 10.1111/j.1754-4505.2008.00003.x. 18271768 10.1111/j.1754-4505.2008.00003.x

[11] Kim BS, Yeo JS, Kim SO. Anesthesia of a dental patient with Angelman syndrome -A case report. Korean J Anesthesiol. 2010;58:207–10. 10.4097/kjae.2010.58.2.207. 20498802 10.4097/kjae.2010.58.2.207 PMC2872855

[12] Gallo C, Marcato A, Beghetto M, Stellini E. Dental treatment in Angelman syndrome patients. 8 case reports. Eur J Paediatr Dent. 2012;13:345–8. 23270298

[13] de Queiroz AM, de Siqueira Melara T, Fernandes Ferreira PD, Lucisano MP, De Rossi A, Nelson-Filho P, Bezerra Silva RA. Dental findings and special care in patients with Angelman syndrome: a report of three cases. Spec Care Dentist. 2013;33:40–5. 10.1111/j.1754-4505.2012.00292.x. 23278148 10.1111/j.1754-4505.2012.00292.x

[14] Zilberman I, Zilberman U. The effect of Angelman syndrome on enamel and dentin mineralization. Spec Care Dentist. 2020;40:574–9. 10.1111/scd.12514. 32881030 10.1111/scd.12514

[15] Schmidt P, Scheiderer M, Suchy L, Schulte A. Einschätzungen zur zahnmedizinischen Versorgung von Personen mit Down-Syndrom in Deutschland durch Eltern — eine qualitative Analyse von Fragebogenangaben. 2024, *79*, 120–132.

[16] Wagner Y, Heinrich-Weltzien R. Evaluation of a regional German interdisciplinary oral health programme for children from birth to 5 years of age. Clin Oral Investig. 2017;21:225–35. 10.1007/s00784-016-1781-8. 26979442 10.1007/s00784-016-1781-8

[17] Wagner Y, Heinrich-Weltzien R. Evaluation of an interdisciplinary preventive programme for early childhood caries: findings of a regional German birth cohort study. Clin Oral Investig. 2016;20:1943–52. 10.1007/s00784-015-1685-z. 26662355 10.1007/s00784-015-1685-z

[18] Schmidt P, Zobel E, Otto R. Etablierung einer Kleinkindsprechstunde Im arbeitsalltag einer Niedergelassenen praxis Zur Kariesprävention Im säuglingsalter. Prophylaxe Impuls. 2019;23:6–13.

[19] Debossan SAT, Deps TD, Prado HV, de Abreu M, Borges-Oliveira AC. Access to oral health care services for individuals with rare genetic diseases affecting skeletal development. Spec Care Dentist. 2022;42:32–40. 10.1111/scd.12639. 34343360 10.1111/scd.12639

[20] Hu K, Da Silva K. Access to oral health care for children with fetal alcohol spectrum disorder: a cross-sectional study. BMC Oral Health. 2022;22. 10.1186/s12903-022-02561-z. 10.1186/s12903-022-02561-z PMC9670607 36384583

[21] Zahran SS, Bhadila GY, Alasiri SA, Alkhashrami AA, Alaki SM. Access to dental care for children with special health care needs: a cross-sectional community survey within jeddah, Saudi Arabia. J Clin Pediatr Dent. 2023;47:50–7. 10.22514/jocpd.2022.032. 36627220 10.22514/jocpd.2022.032

[22] Bernath B, Kanji Z. Exploring barriers to oral health care experienced by individuals living with autism spectrum disorder. Can J Dent Hyg. 2021;55:160–6. 34925516 PMC8641550

[23] Krishnan L, Iyer K, Madan Kumar PD. Barriers to utilisation of dental care services among children with special needs: A systematic review. Indian J Dent Res. 2020;31:486–93. 10.4103/ijdr.IJDR_542_18. 32769288 10.4103/ijdr.IJDR_542_18

[24] da Rosa SV, Moysés SJ, Theis LC, Soares RC, Moysés ST, Werneck RI, Rocha JS. Barriers in access to dental services hindering the treatment of people with disabilities: A systematic review. Int J Dent. 2020;2020:9074618. 10.1155/2020/9074618. 32774378 10.1155/2020/9074618 PMC7396116

[25] Rodriguez Peinado N, Mourelle Martinez MR, Dieguez Perez M, De Nova Garcia MJ. A study of the dental treatment needs of special patients: cerebral paralysis and down syndrome. Eur J Paediatr Dent. 2018;19:233–8. 10.23804/ejpd.2018.19.03.12. 30063157 10.23804/ejpd.2018.19.03.12

[26] AWMF. Caries prevention in permanent teeth - basic recommendations. Register No.: 083– 021;2025. https://register.awmf.org/de/leitlinien/detail/083-021

[27] AWMF. Häusliches mechanisches Biofilmmanagement in der Prävention und Therapie der Gingivitis (S3). *DGZMK-Leitlinie* 2018.

[28] Hennequin M, Allison PJ, Veyrune JL. Prevalence of oral health problems in a group of individuals with down syndrome in France. Dev Med Child Neurol. 2000;42:691–8. 10.1017/s0012162200001274. 11085298 10.1017/s0012162200001274

[29] Schmidt P, Egermann M, Sauerland C, Schulte AG. Caries experience of adults with intellectual disability in the Western part of Germany. J Clin Med. 2021;10:2602. 10.3390/jcm10122602. 34204719 10.3390/jcm10122602 PMC8231577

[30] Schmidt P, Fricke O, Schulte AG. [Outreach dental care for children and adolescents with special Needs - An evaluation of KZBV data]. Gesundheitswesen. 2022;84:952–60. 10.1055/a-1388-7203. 33761559 10.1055/a-1388-7203

[31] Jordan AR, Micheelis W. Fünfte Deutsche mundgesundheitsstudie (DMS V). Köln: Deutscher Zahnärzte; 2016. 10.1186/1472-6831-14-161 PMC4417261 25547464

[32] Descamps I, Marks LA. Oral health in children with down syndrome: parents’ views on dental care in Flanders (Belgium). Eur J Paediatr Dent. 2015;16:143–8. 26147822

[33] Bradley C, McAlister T. The oral health of children with down syndrome in Ireland. Spec Care Dentist. 2004;24:55–60. 10.1111/j.1754-4505.2004.tb01679.x. 15200228 10.1111/j.1754-4505.2004.tb01679.x

[34] Kaye PL, Fiske J, Bower EJ, Newton JT, Fenlon M. Views and experiences of parents and siblings of adults with down syndrome regarding oral healthcare: a qualitative and quantitative study. Br Dent J. 2005;198:571–8. 10.1038/sj.bdj.4812305. discussion 559. 15895058 10.1038/sj.bdj.4812305

[35] Stensson M, Norderyd J, Van Riper M, Marks L, Björk M. Parents’ perceptions of oral health, general health and dental health care for children with down syndrome in Sweden. Acta Odontol Scand. 2021;79:248–55. 10.1080/00016357.2020.1824015. 33017197 10.1080/00016357.2020.1824015

[36] Schulte AG. Salt fluoridation in Germany since 1991. Schweiz Monatsschr Zahnmed. 2005;115:659–62. 16156167

