# Supplementary material for: Oral health care of people with Angelman syndrome in Germany – a questionnaire-based study
Source: BMC Oral Health. 2025 Jun 25;25:959. doi: 10.1186/s12903-025-06357-9 (PMC12199482; doi:10.1186/s12903-025-06357-9)
Supplement: Supplementary file 1 — Supplementary Material 1 [file 12903_2025_6357_MOESM1_ESM.pdf]

supplementary file 1: Questions in english version

A.) sociodemographic aspects	(18 questions)
B.) tooth brushing behavior	(7 questions)
C.) assisted oral health care at home and supportive oral hygiene interventions by dental practitioners	(5 questions)
D.) use of fluoride, at home	(5 questions)
E.) use of dental services (dentist)	(1 question)

A.) sociodemographic aspects:

1.) What is the gender of your family member? ☐ male ☐ female

2.) How old is your family member? _______ years

3.) Does your family member have a legal guardian (= legal carer)?
☐ yes
☐ no, because the family member is not yet 18 years old and the parents have custody.
☐ no, because the family member does not need one

4.) What type of accommodation does your family member live in?
☐ alone
☐ with the parents (at parents' home)
☐ with other family members (at a family member's home)
☐ in supervised living
☐ in a shared accommodation
☐ with a partner
☐ other:____

5.) Since when has your family member mainly lived in Germany?
☐ since birth
☐ since the year: ____________

6.) Does your family member have
☐ German citizenship
☐ Austrian nationality
☐ Swiss nationality
☐ an unlimited residence permit
☐ a temporary residence permit

7.) In which federal state / country does your family member live?
☐ Baden-Württemberg ☐ Bavaria ☐ Berlin ☐ Brandenburg ☐ Bremen
☐ Hamburg ☐ Hesse ☐ Mecklenburg-Western Pomerania
☐ Lower Saxony ☐ North Rhine-Westphalia ☐ Rhineland-Palatinate
☐ Saarland ☐ Saxony ☐ Saxony-Anhalt
☐ Schleswig-Holstein ☐ Thuringia
☐ my family member lives in Switzerland
☐ my family member lives in Austria
region/area/ federal state: ____

8.) Which health insurance company is your family member insured with?
☐ AOK ☐ Barmer (formerly Barmer GEK) ☐DAK
☐ Techniker Krankenkasse ☐ Miners' health insurance fund

☐ company health insurance fund
☐ private health insurance company
☐ other health insurance company, namely: ________________________________

9.) In which country were the parents of your family member born?
Mother: ☐ in Germany ☐ in another country, namely ________________
Father: ☐ in Germany ☐ in another country, namely ________________

10.) Housing/living situation of your family member:
a.) For pre-school children:
Your family member
☐ does not attend kindergarten
☐ attends a kindergarten
☐ attends a special kindergarten
☐ is only cared for at home or in a residential facility
☐ does not apply
☐ other:____

a.) For school children:
Your family member
☐ does not attend a school
☐ attends a school (type of school, if applicable:______________________________)
☐ attends a special school (if applicable, specialisms of the school:_______________)
☐ is only cared for at home or in a residential facility
☐ does not apply
☐ other:____

11.) Training relationship of your family member::
Your family member
☐ does not (yet) have an apprenticeship
☐ has completed an apprenticeship, if yes, which one: ________________________
☐ is only cared for at home or in a residential facility
☐ does not apply
☐ other:____

12.) Your family member's training centre:
☐ Is or was specifically designed to meet the special needs of people with Angelman syndrome If yes, in what form:____
☐ Is or was not specifically geared towards the special needs of people with Angelman syndrome
☐ does not apply
☐ other:____

13.) Your family member's workplace:
☐ is specifically designed to meet the special needs of people with Angelman syndrome If yes, in what form: ____
☐ is not specifically geared towards the special needs of people with Angelman syndrome
☐ does not apply

14.) Your family member's workplace:
☐ is a special workshop for people with disabilities
☐ is a workplace from the primary labour market
☐ does not apply
☐ other:____

15.) Which employment situation applies to your family member?
☐ in full-time employment
☐ part-time gainfully employed
☐ other, namely: ____
☐ does not apply

16.) Has your family member been awarded a care level?
☐ the family member has not been awarded a care level
☐ the family member has been awarded a care level, namely:
☐ care level 1 ☐ care level 2 ☐ care level 3 ☐ care level 4 ☐ care level 5 ☐ no statement

17.) Who completed the questionnaire?
☐ one parent
☐ both parents
☐ a family member (non-parent)
☐ a legal guardian
☐ a caregiver
☐ other (free text response)
☐ no statement

18.) How old are you? _______ years

B.) tooth brushing behavior:

1.) Does your family member with Angelman syndrome receive assistance in tooth brushing?
☐ brushes teeth alone
☐ needs assistance with tooth brushing
☐ receives assistance with tooth brushing
☐ receives assistance with tooth brushing at least once a week
☐ receives assistance with tooth brushing at least once a day

2.) Who assists with tooth brushing?
☐ usually the same person
☐ almost always several persons
☐ does not apply
☐ no statement

3.) How often does your family member with Angel-man syndrome usually brush teeth?
☐ once a day
☐ twice a day
☐ three times a day
☐ after every meal
☐ brushed teeth regularly several times a week
☐ teeth cannot be brushed regularly
☐ no statement

4.) Please estimate how long your family member usually accepts tooth brushing?
☐ less than 1 min
☐ 1–2 min
☐ 2–3 min
☐ this is different every time
☐ no statement

5.) What type of toothbrush does your family member with Angelman syndrome use for tooth brushing?

☐ manual toothbrush
☐ electric toothbrush
☐ sonic electric toothbrush
☐ both manual and electric toothbrush
☐ other:____

6.) Does your family member accept almost all types of toothpaste?
☐ he/she accepts almost all types of toothpaste
☐ he/she only accepts a few types of toothpaste
☐ i don't know/we don't know (anymore)

7.) Are additional dental hygiene implements used apart from the toothbrush?
☐ interdental brushes
☐ dental floss
☐ dental woods
☐ tongue cleaner
☐ oils
☐ no additional implements
☐ other additional implements
☐ no statement

C.) assisted oral health care at home and supportive oral hygiene interventions by dental practitioners:

1.) At what age did tooth brushing start for your family member with Angelman syndrome?
☐ in the first year of life
☐ at the age of 1 year
☐ at the age of 2 years
☐ at the age of 3 years
☐ i/we do not remember (anymore)
☐ no statement

2.) When was your family member's first visit to the dentist?
☐ in the first year of life
☐ at the age of 1 year
☐ at the age of 2 years
☐ at the age of 3–5 years
☐ at the age of 6–10 years
☐ i/we do not remember (anymore)
☐ not yet at all
☐ no statement

3.) Were tooth brushing techniques explained and demonstrated to the family member at the dental practice?
☐ yes
☐ no
☐ other:____
☐ no statement

4.) Was tooth brushing practised with your family member at the dental practice you visited?
☐ yes
☐ no
☐ other:____
☐ no statement

5.) Were tooth brushing techniques also ex-plained and demonstrated to the caregiver who assists the PAS with dental care at the dental practice you visited?
- ☐ yes
- ☐ no
- ☐ does not apply, as no supportive dental care is provided
- ☐ other:____________________
- ☐ no statement

D.) use of fluoride at home:

1.) Does your family member use a fluoride mouth rinse to prevent tooth decay?
- ☐ no
- ☐ yes, namely: ____________________ (product)
- ☐ daily
- ☐ once a week
- ☐ every 2 weeks

2.) Does your family member use a special gel with a high fluoride content to prevent tooth decay?
- ☐ no
- ☐ yes, namely: ____________________ (product)
- ☐ once a week
- ☐ every 2 weeks
- ☐ Once a month
- ☐ every 3 months

3.) Does the household in which your family member lives use table salt with fluoride (more precisely: iodised salt with fluoride) when preparing and seasoning food?
- ☐ yes
- ☐ no
- ☐ not applicable as the family member lives in an assisted accommodation

4.) Is it important to you that the toothpastes your family member uses contain fluoride?
- ☐ yes
- ☐ no
- ☐ i don't know/we don't know (anymore)

5.) Did your family member receive fluoride tablets as a child?
- ☐ yes
- ☐ no
- ☐ i/we do not remember (anymore)
- ☐ no statement

E.) use of dental services (dentist):

1.) Why does your family member visit the dentist in the first place?
- ☐ for regular check-ups
- ☐ for regular prophylaxis sessions
- ☐ for regular check-ups in combination with prophylaxis sessions
- ☐ regular sessions to promote cooperation, as it has not been possible to carry out check-ups while awake
- ☐ for complaints
