# Supplementary material for: Oral health care of people with Angelman syndrome in Germany – a questionnaire-based study
Source: BMC Oral Health. 2025 Jun 25;25:959. doi: 10.1186/s12903-025-06357-9 (PMC12199482; doi:10.1186/s12903-025-06357-9)
Supplement: Supplementary file 2 — Supplementary Material 2 [file 12903_2025_6357_MOESM2_ESM.pdf]

**supplementary file 2: Children with Angelman syndrome**

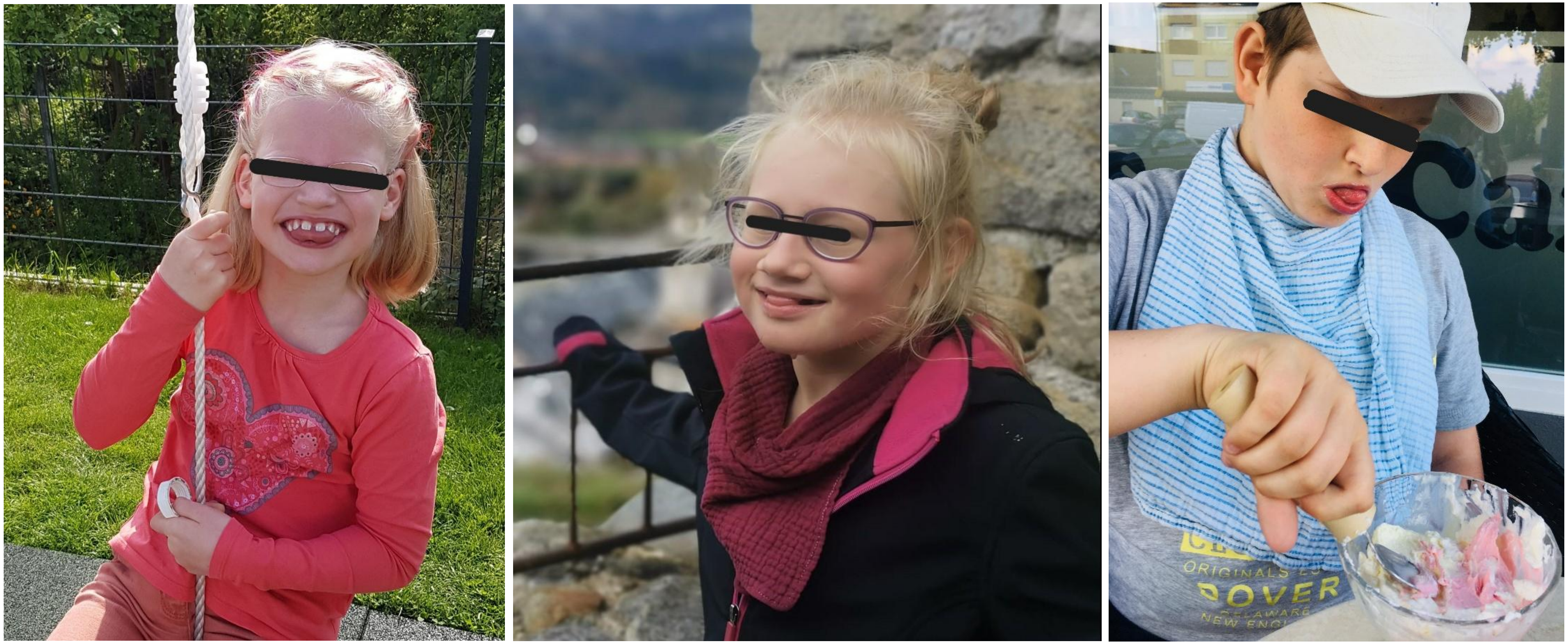

Three photos of children with Angelman syndrome. These photos shows e.g. various syndrome-related manifestations in the orofacial and dental region, such as an elongated, narrow facial shape, mandibular prognathism with potential progeny and a wide mouth, in the sense of macrostomia. Macrostomia is often accompanied by teeth that are both gapped and widely spaced, which, in turn, may be reduced in shape or appear as such.

**Families, who are members of the Angelman e.V. (a German Angelman Syndrome association) have kindly allowed us to use their private photos for this publication. The authors team would like to take the opportunity to thank the families very sincerely!**
